# The ERG1 K^+^ Channel and Its Role in Neuronal Health and Disease

**DOI:** 10.3389/fnmol.2022.890368

**Published:** 2022-05-03

**Authors:** Francisco G. Sanchez-Conde, Eric N. Jimenez-Vazquez, David S. Auerbach, David K. Jones

**Affiliations:** ^1^Department of Pharmacology, University of Michigan Medical School, Ann Arbor, MI, United States; ^2^Department of Pharmacology, State University of New York Upstate Medical University, Syracuse, NY, United States; ^3^Department of Internal Medicine, University of Michigan Medical School, Ann Arbor, MI, United States

**Keywords:** ERG1, *KCNH2*, K_v_11.1, epilepsy, brain, electrophysiology

## Abstract

The ERG1 potassium channel, encoded by *KCNH2*, has long been associated with cardiac electrical excitability. Yet, a growing body of work suggests that ERG1 mediates physiology throughout the human body, including the brain. ERG1 is a regulator of neuronal excitability, ERG1 variants are associated with neuronal diseases (e.g., epilepsy and schizophrenia), and ERG1 serves as a potential therapeutic target for neuronal pathophysiology. This review summarizes the current state-of-the-field regarding the ERG1 channel structure and function, ERG1’s relationship to the mammalian brain and highlights key questions that have yet to be answered.

## Introduction

**FIGURE 1:**
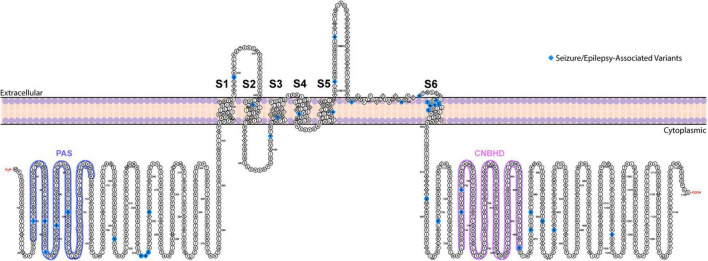
Topological map of ERG1 channel. The schematic depicts one ERG1 subunit with the helical transmembrane domains, S1–S6, labeled. The S5 and S6 helices along with the extracellular S5–S6 linker from each subunit, combine to form the ion conduction pathway. The intracellular side contains the gating regulators: PAS domain (blue) and CNBH domain (purple). Positions of ERG variants that have been linked to seizures and epilepsy are highlighted in blue.

The *KCNH2* gene (also called the *human ether-a-go-go-related gene*) encodes ERG1/K_v_11.1, which forms the voltage-gated potassium channel that conducts I_Kr_ and regulates human cardiac electrical excitability (Sanguinetti et al., 1995; Trudeau et al., 1995). Since it was identified as a major regulator of cardiac excitability, ERG1 has generated considerable scientific interest due to its role in both genetically and pharmacologically linked cardiac arrhythmias (Curran et al., 1995; Sanguinetti et al., 1995). While its physiological function has been mostly characterized in cardiac cells, ERG1 channels are expressed in a wide variety of tissues including the gastrointestinal tract, kidney, pancreas, endocrine tissues, bone marrow, and brain (Warmke and Ganetzky, 1994; Schledermann et al., 2001; Muhlbauer et al., 2007; Sjostedt et al., 2020). The contribution of ERG1 channels in neuronal activity has not been explored to nearly the same extent as the heart, although ERG1 channels are associated with neurological dysfunction such as schizophrenia and epilepsy (Pessia et al., 2008; Omichi et al., 2010; Tu et al., 2011; Zamorano-Leon et al., 2012; Li et al., 2016; Zarroli and Querfurth, 2018; Figure 1 and Table 1). This review will summarize the current state-of-the-field regarding the ERG1 channel in the mammalian brain, highlighting what is known and key questions remaining to be answered.

**TABLE 1:** Summary of seizure/epilepsy-associated *KCNH2* variants.

Variants	Region
I30	PAS
A57	PAS
C64	PAS
R73	PAS
Y99	PAS
A193	N-terminus
P241	N-terminus
R242	N-terminus
S243	N-terminus
R252	N-terminus
A429	S1–S2 loop
D456	S2 segment
Y493	S2–S3 loop
D501	S3 segment
A558	S5 segment
G572	S5–S6 linker
R582	S5–S6 linker
T613	P-loop
N629	P-loop
N633	P-loop
F640	S6 segment
I642	S6 segment
C643	S6 segment
V644	S6 segment
M645	S6 segment
S649	S6 segment
Q676	C-terminus
E678	C-terminus
R744	CNBHD
G749	CNBHD
R863	CNBHD
P872	C-terminus
E876	C-terminus
Q901	C-terminus
G916	C-terminus
G1036	C-terminus

*The numbers indicate the position of each amino acid in the ERG1 channel.*

## ERG1 Structure and Function

### Structure

ERG1, like other voltage-gated potassium channels, show a 4-fold tetrameric structure containing a voltage sensor (helices S1–S4) and a pore-forming region (S5–S6). ERG1 channels, members of the broader KCNH channel family, have a cytosolic Per-Arnt-Sim (PAS) domain and a cytosolic cyclic nucleic binding homology domain (CNBHD) that interact directly to regulate channel gating (Figure 1; Warmke and Ganetzky, 1994; Morais Cabral et al., 1998; Sanguinetti and Tristani-Firouzi, 2006; Tao et al., 2010; Gustina and Trudeau, 2011; Whicher and MacKinnon, 2016; Harley et al., 2021).

**FIGURE 2:**
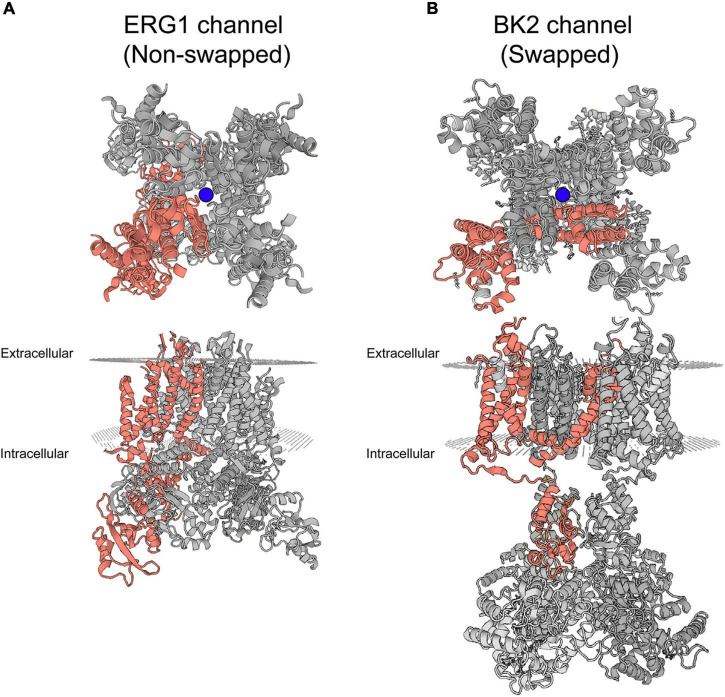
Swapped and non-domain swapped structure in K_v_ channels. **(A)** Cryo-EM structure of ERG1 (PBD: 5va3) displayed from the membrane plane (top) and the cytoplasmic side (bottom) depicts a non-domain-swapped architecture. The voltage sensing domains contact the pore helices from the same subunit. **(B)** Cryo-EM structure of the BK2 channel (PBD: 5wie) displayed from the membrane plane (top) and the cytoplasmic side (bottom) depicts the domain-swapped architecture identified in the majority of K_v_ channels. Peripheral voltage sensing domains contact the pore helices of the neighboring subunit. One complete subunit is colored as shown. The blue balls represent the location of K^+^ ions at the selectivity filter.

Recent cryo-EM structures of ERG1 provided exciting insight into the basic structure of ERG1 channels. The most notable feature is the positioning of each voltage sensing domain directly against the pore-forming helices (S5 and S6) of their own subunit in ERG1 (Figure 2A; Wang and MacKinnon, 2017; Asai et al., 2021). A very short S4–S5 linker sequence functions as a ligand, binding to the C-terminal end of the inner pore S6 helix below the activation gate, and stabilizes the closed state at rest (Whicher and MacKinnon, 2016; Malak et al., 2017, 2019; Wang and MacKinnon, 2017). This stands in contrast to the broader family of voltage gated potassium channels, which display a domain-swapped voltage sensing domain (Figure 2B).

Two potential accessory subunits, KCNE1 and KCNE2, have been suggested to interact with the pore-forming ERG1 subunit (McDonald et al., 1997; Isbrandt et al., 2002; Abbott et al., 2007). KCNE1 and KCNE2 are single transmembrane helices with extracellular N-termini and cytoplasmic C-termini (Abbott and Goldstein, 2001). Originally reported as modifiers of the KCNQ1 channel (Takumi et al., 1991; Melman et al., 2001, 2004; Panaghie et al., 2006; Chen et al., 2009; Goldman et al., 2009; Strutz-Seebohm et al., 2011), KCNE1 and KCNE2 expression alters ERG1 gating kinetics and protein degradation in native cardiac tissue, as well as heterologous expression systems (Yang et al., 1995; Bianchi et al., 1999; Mazhari et al., 2001; Ackerman et al., 2003; Zhang M. et al., 2012). Both subunits are expressed in the mammalian brain along with KCNE3–KCNE5 (Lundquist et al., 2006; Sjostedt et al., 2020), where they could act to modify ERG1 function. The specific role of these accessory subunits in native ERG1 function is still of debate.

### Alternate Transcripts, Splice Variants, and Subunits

**FIGURE 3:**
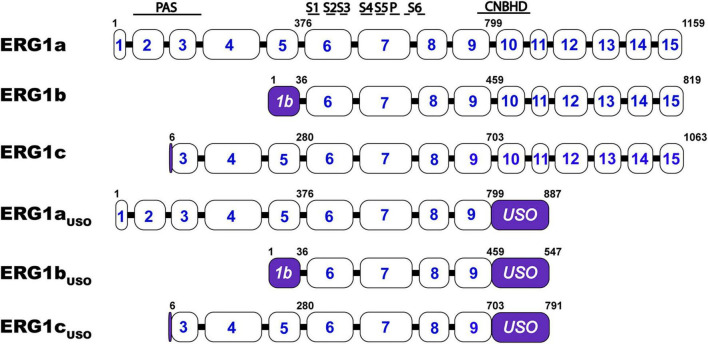
Schematic comparison of the ERG1 isoforms. *KCNH2* exon structure encoding ERG1a, ERG1b, ERG1c, ERG1a_USO_, ERG1b_USO_, ERG1c_USO_. Approximate structural correlates of the transmembrane, PAS, and CNBH domain positions are shown at top. The numbers inside the boxes represent the *KCNH2* gene’s coding exons. The numbers above the illustrations represent the position of the residues that define each variant. The regions that identified each splicing isoform are highlighted in purple.

*KCNH2* encodes multiple ERG1 distinct transcripts that, when transcribed, combine to form hetero-tetrameric ERG1 channels: ERG1a, ERG1b, ERG1c, and ERG1_USO_ (Figure 3). All ERG1 subunits share an identical transmembrane core but vary in the length and structure of their N- and C- termini. ERG1a was the first subunit identified and the most studied, however, all five transcripts have been shown to modify ERG1 currents. ERG1a, ERG1b, and ERG1c transcription is initiated from distinct subunit-specific promoter regions making them alternate transcripts of the same gene (Lees-Miller et al., 1997; London et al., 1997; Huffaker et al., 2009). Conducting ERG1 channels of native tissue can comprise at least three subunits: ERG1a, ERG1b, and ERG1c (Lees-Miller et al., 1997; London et al., 1997; Jones et al., 2004, 2014; Huffaker et al., 2009; Calcaterra et al., 2016; Carr et al., 2016; Goversen et al., 2019). 15 exons encode the ERG1a transcript, the first five of which encode the long N-terminal domain that contains the channel’s PAS domain.

In ERG1b, a transcription start site in intron 5 encodes a single 1b-specific exon that replaces exons 1–5 and encodes a shorter, PAS-deficient N-terminal domain (London et al., 1997; Splawski et al., 1998). It was also demonstrated that ERG1a and ERG1b preferentially interact and co-assemble in the ER to form heterotetrameric ion channels at the plasma membrane in cardiac tissue and human stem cell-derived cardiomyocytes (Kagan et al., 2000; Jones et al., 2004; Sale et al., 2008; McNally et al., 2017). Additional work in human heart demonstrated that individual ERG1a and ERG1b mRNA transcripts co-assemble to promote channel translation, thereby enhancing ERG1 surface expression and membrane currents (Eichel et al., 2019).

A third transcription start site within intron 2 initiates a third ERG1 subunit, ERG1c (KCNH2-3.1). ERG1c lacks the first two ERG1a exons and contains an extended exon 3. Thus, the ERG1c subunit lacks the first 102 amino acids of ERG1a and displays electrophysiological properties similar to ERG1b. ERG1c is associated with cognitive dysfunction (Huffaker et al., 2009; Calcaterra et al., 2016; Carr et al., 2016). ERG1a and ERG1b are expressed in both heart and brain, whereas ERG1c appears to be largely limited to the mammalian brain, particularly in the hippocampus (Warmke and Ganetzky, 1994; Saganich et al., 2001; Guasti et al., 2005; Huffaker et al., 2009; Uhlen et al., 2015; Carr et al., 2016; Ren et al., 2020).

The *KCNH2* splice variant, ERG_USO_, is generated by alternative splicing leading to an additional 88-amino acids extending from exon 9 that then ends with a 3′ UTR in intron 9, thereby omitting the final six *KCNH2* exons. ERG_USO_ channels can generate from any of the three N-terminal variants (ERG1a_USO_, ERG1b_USO_, ERG1c_USO_) and form non-conducting channels that may play a role in regulating ERG1 current density (Kupershmidt et al., 1998; Guasti et al., 2008; Gong et al., 2010). Future studies may identify additional ERG1 splice variants, as the role of *KCNH2* in tissues outside the brain and heart is investigated. Finally, despite improved understanding of ERG1 subunit heteromerization, the native ERG1 subunit stoichiometry *in vivo* has yet to be determined.

### ERG1 Gating

**FIGURE 4:**
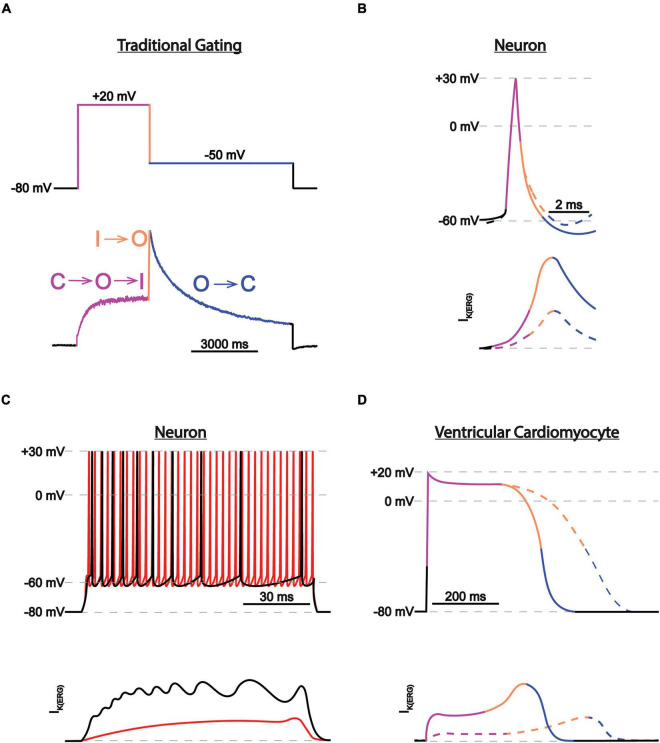
ERG1 currents in heart vs. brain. **(A)** Current trace during a two-step voltage protocol. The transitions between states are color-coded and correspond with the transitions between the closed (C), activated (A), and inactivated (I) states. **(B)** Depiction of individual neuronal action potential and corresponding I_K(ERG)_ before (*solid lines*) and after (*dotted lines*) I_K(ERG)_ reduction. Traces are color-coded as in **(A)**. I_K(ERG)_ reduction does not affect action potential shape but depolarizes the resting membrane potential, moving the cell closer to firing threshold. **(C)** Depiction of neuronal action potential trains and corresponding I_K(ERG)_ before (*black*) and after (*red*) I_K(ERG)_ reduction. I_K(ERG)_ reduction accelerates action potential firing and inhibits spike frequency adaption during prolonged stimuli. **(D)** Depiction of individual cardiac action potential and corresponding I_Kr_, conducted by ERG1 before (*solid lines*) and after (*dotted lines*) I_K(ERG)_ reduction. Traces are color-coded as in **(A)**. I_K(ERG)_ reduction prolongs the cardiac action potential.

ERG1 channels, like other voltage-gated channels, transition between closed, open, and inactivated states in response to changes in the membrane electric field. It is the concerted movement of the four voltage sensing domains that drives the conformational changes that open (activate) or close (deactivate) the pore (Hodgkin and Huxley, 1952; Armstrong and Bezanilla, 1973; Bezanilla et al., 1982; Piper et al., 2005; Goodchild and Fedida, 2014; Dou et al., 2017). ERG1 activation and deactivation are unusually slow, occurring over hundreds of milliseconds to whole seconds. In contrast, ERG inactivation, which is believed to be a C-type mechanism at the selectivity filter, is remarkably fast (≤10 ms). Thus, activated ERG channels inactivate almost immediately (Kiehn et al., 1996, 1999; Rasmusson et al., 1998). It is this disparate time course of ERG activation/deactivation vs. inactivation that gives rise to the unique current profile of the ERG channel family (Figure 4). During depolarization ERG channels quickly transition into their inactivated state, thereby suppressing current at depolarized potentials. Upon repolarization, ERG channels quickly recover from the inactivated state, but because of the slow deactivation time course, the channels remain in a conductive state.

While the role of the voltage sensing domain is somewhat clear, how voltage sensing domain movements translate to pore opening/closing is still a topic of discussion. ERG1 cryo-EM structures, along with homology models with KcsA, Shaker, Eag1, K_v_AP, and K_v_1.2 have provided excellent insight into ERG1 channel structure and function. Most K_v_ channels have a long helical S4–S5 linker that allows the VSD from one subunit to interact with the pore domain from the adjacent subunit – a domain-swapped configuration (Long et al., 2005, 2007). The S4–S5 linker of ERG1 (as well as other KCNH, cyclic nucleotide-gated, and K_Ca_ channels) is short, tethering the VSD to the pore domain of the same subunit – a non-domain swapped configuration. The positioning of the ERG voltage sensor helps to explain the interesting finding that splitting ERG1 channels at the S4–S5 linker, which resides between the voltage-sensing and pore-forming domains, still produces functional voltage-dependent channels (Lörinczi et al., 2015; de la Peña et al., 2018a,b). These data suggest that the voltage-sensing domains of ERG1 and other non-domain swapped channels couple with the pore through direct interactions between the transmembrane helices, rather than through the S4–S5 linker as seen in Shaker-like potassium channels (Lu et al., 2002; Whicher and MacKinnon, 2016; Hite and MacKinnon, 2017; Wang and MacKinnon, 2017).

The N-terminal PAS domain and the C-terminal CNBHD tightly regulate the kinetics and voltage dependence of ERG channel activation and inactivation. In ERG1a, the PAS domain “docks” with the C-terminal CNBHD, allowing the N-terminal PAS cap to interact directly with the S4–S5 linker. The CNBHD is homologous to the cyclic nucleotide domains of nucleotide gated channels (Guy et al., 1991; Warmke et al., 1991; Zagotta et al., 2003), but the CNBHD of ERG1 is not bound by cyclic nucleotides (Robertson et al., 1996; Brelidze et al., 2009). Instead, amino acids immediately distal to the ERG1 CNBHD (860–862) form an “intrinsic ligand” that binds to the CNBHD at the predicted cyclic nucleotide binding pocket (Brelidze et al., 2012, 2013; Codding and Trudeau, 2019). Disrupting the ERG1 intrinsic ligand disrupts the PAS/CNBHD interaction (Codding and Trudeau, 2019). Disrupting the interaction between the PAS and CNBHD significantly accelerates activation, deactivation, and inactivation recovery (Gustina and Trudeau, 2009, 2012; Gianulis and Trudeau, 2011; Codding and Trudeau, 2019). ERG1b and ERG1c lack fully functional N-terminal domains. As a result, the activation and deactivation kinetics of ERG1a are slow in comparison (Sanguinetti et al., 1995; Trudeau et al., 1995; Wang et al., 1997a). ERG1b and ERG1c homomers have dramatically faster activation, deactivation, and inactivation recovery kinetics (Saenen et al., 2006; Larsen et al., 2008; Larsen and Olesen, 2010; Trudeau et al., 2011; Heide et al., 2012); making the PAS-deficient subunits major promoters of inactivation recovery (Perissinotti et al., 2018).

## ERG1 in Neuronal Physiology and Pathophysiology

### Neuronal Distribution

**FIGURE 5:**
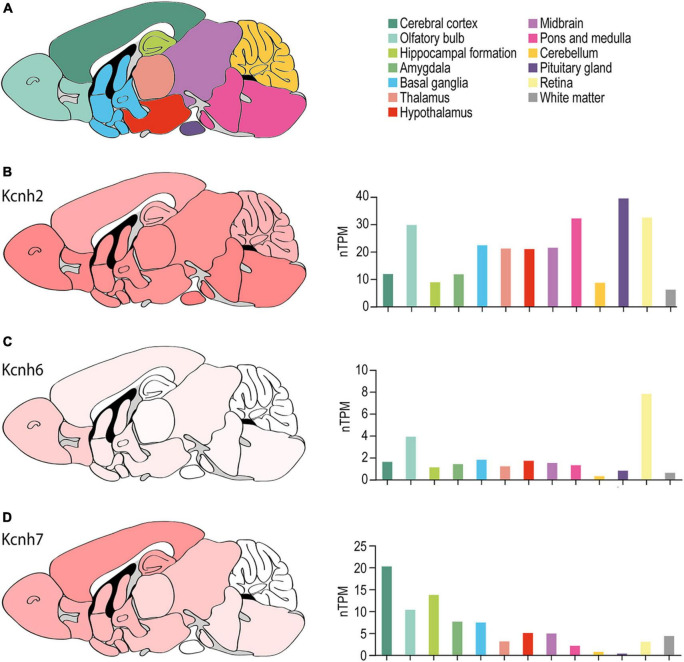
ERG channel distribution in mouse brain. **(A)** Color-coded schematic depicting a sagittal cross section of the human brain. **(B–D)** Regional mouse brain heat maps (*left*) and RNA expression levels (*right*) for *Kcnh2* **(B)**, *Kcnh6* **(C)**, and *Kcnh7* **(D)**. Individual bars are color-coded to correspond with regions highlighted in **(A)**. nTPM, normalized protein-coding transcripts. Adapted Human Protein Atlas images and data available from v21.proteinatlas.org/ENSG00000055118-KCNH2/ brain, v21.proteinatlas.org/ENSG00000173826-KCNH6/brain, and v21.proteinatlas.org/ENSG00000184611-KCNH7/brain.

In addition to ERG1, the brain expresses two additional ERG orthologs: ERG2 and ERG3, encoded by *KCNH6* and *KCNH7*, respectively (Warmke and Ganetzky, 1994; Shi et al., 1997; Wymore et al., 1997; Papa et al., 2003; Polvani et al., 2003; Guasti et al., 2005). All three orthologs display region and neuronal cell type-specific distributions (Warmke and Ganetzky, 1994; Chiesa et al., 1997; Wymore et al., 1997; Saganich et al., 2001; Papa et al., 2003; Figure 5).

#### The Murine Brain

ERG1 expression has been reported throughout the murine brain (Figure 5). ERG3 expression is similar to ERG1 but displays enrichment in the cerebral cortex and is nearly absent from the cerebellum (Figure 5D). ERG1 and ERG3 transcripts were reported in layers II through VI of the cerebral cortex, but ERG1 transcript levels are generally lower compared to ERG3 (Saganich et al., 2001; Guasti et al., 2005). In the hippocampus, Saganich et al. (2001) reported ERG1 and ERG3 transcripts at moderate-to-high levels in CA1 pyramidal cells and dentate gyrus cells. Interestingly, ERG1 mRNA and protein was enriched and ERG3 absent in inhibitory interneurons of the hippocampus and cerebral cortex (Saganich et al., 2001). Subsequent studies reported ERG1 and ERG3 transcripts and protein in CA1, CA2, and CA3 pyramidal cells in rat (Papa et al., 2003; Guasti et al., 2005). There was low to moderate expression of ERG1 or ERG3 in the basal ganglia, the thalamus, the hypothalamus, the olfactory bulb, and low expression in the amygdala. Lastly, ERG1 and ERG 3 were also highly expressed throughout the midbrain, the vestibular nuclei, and the Purkinje cell layer and the deep nuclei of the cerebellum (Saganich et al., 2001; Guasti et al., 2005).

Saganich et al. (2001) reported that ERG2 transcripts were exclusively seen within the mitral cell and periglomerular layers of the olfactory bulb (Figure 5C). Later studies reported similar findings, where ERG2 transcripts were enriched in the olfactory bulb, but also reported low ERG2 transcript levels in areas previously thought to be ERG2-deficient, including the cerebral cortex, hippocampus, hypothalamus, thalamus, and brainstem (Papa et al., 2003; Polvani et al., 2003; Guasti et al., 2005). Outside of the olfactory bulb, murine ERG expression is predominantly ERG1 and ERG3 (Papa et al., 2003; Polvani et al., 2003; Guasti et al., 2005).

#### The Human Brain

**FIGURE 6:**
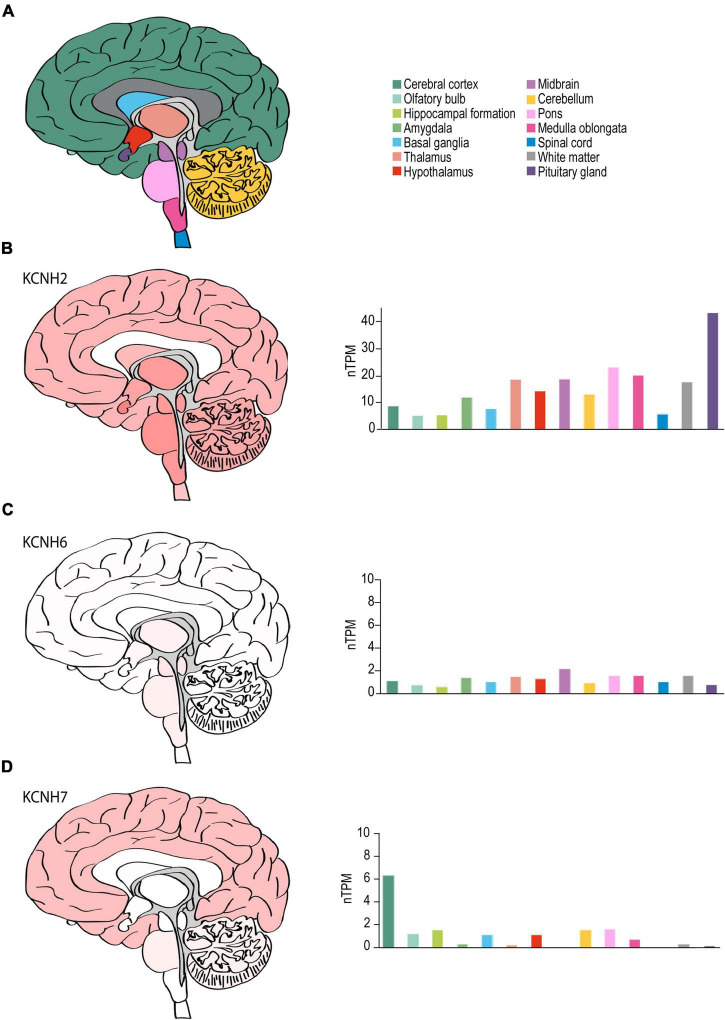
ERG channel distribution in human brain. **(A)** Color-coded schematic depicting a sagittal cross section of the human brain. **(B–D)** Regional human brain heat maps (*left*) and RNA expression levels (*right*) for *KCNH2* **(B)**, *KCNH6* **(C)**, and *KCNH7* **(D)**. Individual bars are color-coded to correspond with regions highlighted in **(A)**. nTPM, normalized protein-coding transcripts. Adapted Human Protein Atlas images and data available from v21.proteinatlas.org/ENSG00000055118-KCNH2/brain, v21.proteinatlas.org/ENSG00000173826-KCNH6/brain, and v21.proteinatlas.org/ENSG00000184611-KCNH7/brain.

Although *KCNH2* was first identified out of a human hippocampus cDNA library (Warmke and Ganetzky, 1994), data on the distribution of *KCNH2* and ERG1 in the human brain is limited. RNA-seq data from human brain tissue has shown that, like the mouse, *KCNH2* mRNA shows limited regional specificity. The one exception is in the pituitary gland, which displays a nearly 2-fold enrichment over the next highest region, the pons (43.6 nTPM vs. 22.4 nTPM, respectively) (Figure 6). The most notable differences between the human and mouse are that *KCNH2* levels are substantially lower in the human olfactory bulb and retina compared to the mouse. *KCNH7* is enriched in the human cerebellum and found to a lesser degree in the olfactory bulb, hippocampus, basal ganglia, hypothalamus, like it is in mouse. In contrast to the mouse, *KCNH7* transcripts are present in the human cerebellum and absent from the midbrain and thalamus (Figure 6D). Unlike in mouse, *KCNH6* expression is not enriched in the human olfactory bulb, but is somewhat upregulated in the thalamus, midbrain, and brain stem. And like its murine counterpart, *KCNH6* is relatively absent in the human cerebral cortex (Figure 6C). It is worth noting that the abundance of ERG1-encoding transcripts is higher than transcripts encoding either ERG2 or ERG3 in both mouse and human.

### ERG1 in Neuronal Excitability

#### ERG1 Is a Major Component of I_K(ERG)_

**FIGURE 7:**
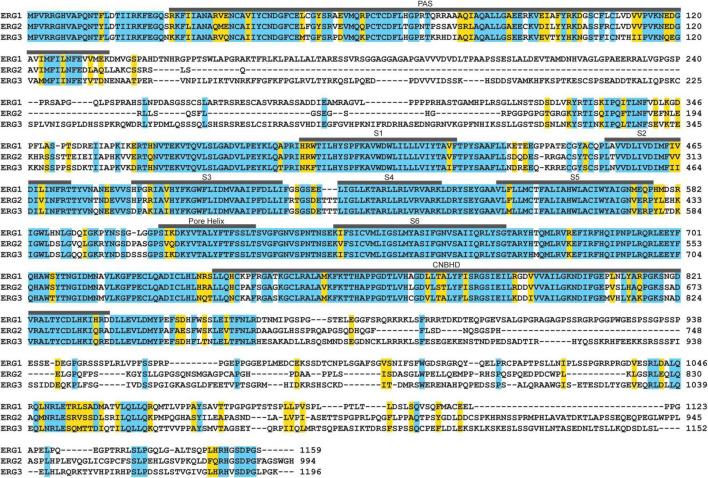
ERG ortholog alignment. ERG1, ERG2, and ERG3 channels display high amino acid sequence homology in their transmembrane segments, pore helix, the PAS and CNBH domains. Residues identical between all three are highlighted in blue, conserved residues are highlighted in yellow.

ERG1, ERG2, and ERG3 collectively conduct neuronal ERG current [I_K(ERG)_] (Shi et al., 1997; Papa et al., 2003; Polvani et al., 2003; Guasti et al., 2005). Defining ERG1-specific contributions to neuronal physiology has been challenging. The three ERG orthologs display very high sequence homology (Figure 7 and Table 2) and similar pharmacological profiles, hampering attempts to isolate ERG1-specific currents (Shi et al., 1997; Wimmers et al., 2001, 2002). Additionally, the overlapping expression profiles of ERG1 and ERG3 *in vivo* (Figures 5, 6), combined with electrophysiological studies co-expressing the two (Wimmers et al., 2001, 2002) suggest that ERG1 and ERG3 form heterotetrameric channels. Thus, the individual contribution of ERG1 in most cases of neuronal physiology and pathophysiology remains unclear.

**TABLE 2:** ERG ortholog sequence homology.

	ERG1 × ERG2	ERG1 × ERG3	ERG2 × ERG3
Sequence identity	52.79%	55.44%	50.41%
Identical residues	644	688	609
Conserved residues	151	240	200

**FIGURE 8:**
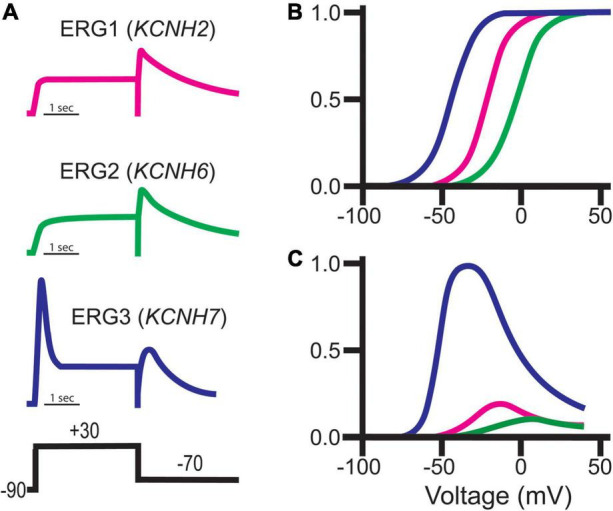
ERG channel family gating. **(A)** Schematic representations of typical ERG currents obtained using the putative pulse protocol displayed at bottom. **(B)** Representative normalized Boltzmann curves depicting the voltage-dependent activation for ERG1 (*magenta*), ERG2 (*green*), and ERG3 (*blue*). **(C)** Representative steady-state conductance curves for ERG1 (*magenta*), ERG2 (*green*), and ERG3 (*blue*). Steady-state conductance curves are depicted relative to ERG3 to highlight the impact of the distinct ERG gating on conductance. Images adapted from Shi et al. (1997). Copyright 1997 Society for Neuroscience.

Despite their sequence similarities, ERG orthologs do display subtle differences in their voltage-dependence and kinetic properties, which has provided insight into brain region-specific ERG ortholog contributions (Figure 8). ERG1 channels activate around −50 mV, whereas ERG3 and ERG2 channels activate at −70 and −40 mV, respectively (Shi et al., 1997; Wimmers et al., 2001, 2002; Sturm et al., 2005; Figure 8B). The peak conductance/voltage curves of each ortholog follow their respective voltage dependence of activation (Figure 8C). In line with their relatively negative voltage dependence, ERG3 channels also activate at a much faster rate compared to ERG1 and ERG2. Interestingly, while the time course of ERG1 and ERG3 activation is best fitted with a single exponential function, the activation time course of ERG2 channels is best described with a double exponential.

All three orthologs inactivate, which suppresses current following activation. Unlike ERG1 and ERG2, which inactivate rapidly, ERG3 channels inactivate at a much slower rate. This relatively slow inactivation time course, coupled with a hyperpolarized voltage dependence of activation, results in a significant increase in overall ERG3 conductance during depolarization compared to ERG1. Following that same trend, ERG2’s activation at more positive potentials results in less ERG2 conductance compared to ERG1 (Shi et al., 1997; Schledermann et al., 2001; Figure 8). The somewhat distinct biophysical profile of ERG3 has been used to predict when it is the dominant ERG ortholog, however, this technique is problematic due differences in recording conditions and potential unknown ERG channel modifiers.

#### I_K(ERG)_ Regulates Neuronal Excitability

The ERG family of channels regulates neuronal action potential firing frequency, spike frequency adaptation, and resting membrane potential, but have a limited role in shaping individual action potential shape (Chiesa et al., 1997; Gong et al., 2010; Calcaterra et al., 2016). Slow ERG deactivation allows channels to accumulate in their conductive state during repetitive firing, which diminishes electrical activity during prolonged stimuli. The increase in potassium conductance hyperpolarizes the resting membrane potential and counteracts sodium flux, thereby lowering spike frequency or even terminating a burst (Chiesa et al., 1997; Figures 4B,C). Interestingly, similar ERG current characteristics are observed in adult murine cardiomyocytes, which elicit action potentials at rates of up to 14 Hz or 840 bpm (London et al., 1997).

The role of ERG channels on neuronal firing contrasts with their role in human cardiac tissue, where ERG1 channels regulate the shape and duration of each individual action potential (Sanguinetti et al., 1995; Trudeau et al., 1995). During cardiac depolarization, ERG1 channels activate and then quickly inactivate. The dynamic equilibrium between the activated and inactivated states then mediates the duration and shape of the cardiac action potential plateau, whereby disrupting inactivation hastens repolarization (Zhang H. et al., 2012; Harley et al., 2016; Yu et al., 2016). Upon repolarization ERG1 channels recover from inactivation but close slowly, triggering a surge of ERG1 conductance that terminates the action potential. Slow pore closure also prolongs the cardiac refractory period by allowing a slowly diminishing potassium conductance following repolarization (Cheng and Claydon, 2012; Shi et al., 2020b; Figure 4D).

To date, a single study selectively highlights ERG ortholog-specific behavior in neurons (ERG3). Xiao et al. (2018), showed that shRNA-mediated ERG3 knockdown in mouse hippocampal CA1 pyramidal neurons and dentate gyrus granule cells led to enhanced excitability and seizure susceptibility. They also demonstrated that an ERG activator protected against epileptogenesis and that ERG3 expression is reduced in hippocampal epileptic foci. These data begin to make pre-clinical connections between ERG channels and electrical dysfunction in the brain.

Pharmacological I_K(ERG)_ block, which blocks all three orthologs, promotes hyperexcitability in neuronal populations throughout the murine brain. ERG block increased firing frequency and reduced spike frequency adaptation in midbrain dopamine neurons (Ji et al., 2012), L5 neocortical pyramidal neurons (Cui and Strowbridge, 2018, 2019), medial vestibular neurons (Pessia et al., 2008), mitral/tufted neurons (Hirdes et al., 2009), and cells in the medial nucleus of the trapezoid body (MNTB; Hardman and Forsythe, 2009). Multiple studies reported depolarized resting membrane potentials following selective I_K(ERG)_ block (Hardman and Forsythe, 2009; Hirdes et al., 2009).

ERG channels have also been implicated in sensory homeostatic feedback mechanisms. Sensory neurons in the basal layer of the mouse vomeronasal organ demonstrate dynamic control over inputs and outputs via regulating ERG expression. These relationships in tandem with ERG’s role in firing result in a system that adjusts stimulus-response relationships in a targeted use-dependent and layer-specific manner (Hagendorf et al., 2009).

Functional ERG channels have also been discovered in murine mitral/tufted cells of the olfactory bulb, implying that they may play a role in regulating excitability in a variety of sensory organs (Hirdes et al., 2009). Similarly, it was discovered that ERG channels can influence the resonance properties of medial vestibular nucleus neurons, regulating excitability and discharge dynamics (Pessia et al., 2008). During the antenatal development of the spinal network, ERG expression has been shown to play an important role in the control of GABAergic interneuron excitability (Furlan et al., 2007). ERG activity appears to modulate excitability and epinephrine release in chromaffin cells, which is a fundamental neurotransmitter shaping cardiac function and links LQT and catecholaminergic signaling (Gullo et al., 2003). There is also evidence that ERG channels play an important role in limiting excitability and minimizing depolarization inactivation in dopaminergic neurons in the midbrain. This finding is intriguing because ERG blockers may have therapeutic benefits for psychiatric diseases associated with dopamine signaling defects (Ji et al., 2012). ERG currents in the medial nucleus of the trapezoid body of the auditory brainstem can also supplement K_v_1 currents in limiting AP firing at around threshold voltages; in fact, ERG may play a special role during periods of high activity when K^+^ concentration is high (Hardman and Forsythe, 2009). Thus, the presence of ERG within the auditory pathway raises intriguing links with LQT2, implying that ERG dysfunction in the brainstem may underpin the auditory trigger of LQT2 cardiac events via auditory hyperexcitability and exaggerated startle reflexes (Hardman and Forsythe, 2009). Additionally, ERG plays a role in slow afterhyperpolarizations in neurons of the substantia nigra compacta (Nedergaard, 2004).

#### ERG1 in I_K(ERG)_

Despite the limitations in identifying ERG1-specific contributions, several studies have evaluated the collective contribution of ERG channels in neuronal function using selective pharmacological block of I_K(ERG)_. When these studies are examined in the context of *in situ* hybridization data (Saganich et al., 2001; Papa et al., 2003), immunohistochemistry data (Papa et al., 2003; Guasti et al., 2005), and the most recent RNA-seq data (Figures 5, 6), we can form hypotheses regarding the relative contribution of ERG1 to I_K(ERG)_ in select cell types.

ERG1 is likely a major regulator of interneuron excitability. Selective I_K(ERG)_ block increased action potential firing frequency, reduced first spike latency, increased the number of complex spike spikelets, and inhibited spike frequency adaptation in cerebellar GABAergic Purkinje cells of neonatal and adult mice (Sacco et al., 2003). The same study reported I_K(ERG)_ voltage dependence comparable to ERG1 channels (Sacco et al., 2003). Other work in mouse cerebellar Purkinje cells reported I_K(ERG)_ characteristics similar to ERG1 homomers and ERG1/ERG3 concatemers (Niculescu et al., 2013). Niculescu et al. (2013) also demonstrated that mGluR1 activation inhibited neuronal I_K(ERG)_. Finally, Furlan et al. (2005, 2007) reported transient ERG1/3-like I_K(ERG)_ current in the GABAergic interneurons of the ventral horn from developing mice, suggesting a role in axonal guidance and network formation. Combined with mRNA and protein data that demonstrated targeted ERG1 expression in interneurons, these biophysical data suggest a key role for ERG1 in interneuron excitability and function.

Thryotropin-releasing hormone was initially shown to inhibit I_K(ERG)_ currents in rat pituitary cells in 1990 (Bauer et al., 1990). The same group went on to elegantly demonstrate that ERG currents regulate the resting membrane potential as well as the onset and frequency of action potential firing in lactotrophs (Bauer, 1998; Bauer et al., 1999; Schafer et al., 1999; Schwarz and Bauer, 1999), and ultimately that ERG block increased prolactin secretion (Bauer et al., 1999). Later work demonstrated that ERG1-encoding transcripts are the dominant transcript in the pituitary gland (Figure 5). Together these data strongly suggest that ERG1 is the primary contributor to I_K(ERG)_ in the pituitary gland.

Astrocytes work to reduce local potassium accumulation during high neuronal activity (Hertz, 1965; Orkand et al., 1966; Bellot-Saez et al., 2017). Impaired potassium buffering is associated with epilepsy (David et al., 2009), cortical spreading depression (Somjen, 2002), and cell death (Leis et al., 2005). Emmi et al. (2000) demonstrated that cortical astrocytes from rat selectively express ERG1, not ERG2 or ERG3. They also demonstrated that pharmacological I_K(ERG)_ block resulted in impaired potassium clearance in cultured astrocytes, suggesting that ERG1-mediated I_K(ERG)_ plays a vital role in the spatial buffering process (Emmi et al., 2000).

The roles of ERG1 channel subunit composition are only just beginning to be explored. ERG1c is upregulated relative to ERG1a in the hippocampus of schizophrenic patients (discussed below) (Huffaker et al., 2009). It was also shown that transforming the heteromeric gating phenotype of native heteromeric ERG1a/1b channels to a homomeric ERG1a-like phenotype is arrhythmogenic in cardiac tissue (Harley et al., 2016). These findings demonstrate that channel composition is equally as important as channel abundance in excitable tissue. ERG1 channel subunit composition and its impact on ERG channel kinetics could represent a physiological mechanism employed to fine tune ERG currents to suit the need of their cellular environment.

#### ERG1 in Schizophrenia

The neuronal ERG1 splice variant, ERG1c (aka KCNH2-3.1), is associated with schizophrenia (Huffaker et al., 2009; Atalar et al., 2010). Under healthy conditions, ERG1c expression is low due to poor trafficking and only rescuable by proteosome inhibition (Calcaterra et al., 2016). However, ERG1c is upregulated in hippocampal brain tissue from schizophrenic patients (Huffaker et al., 2009). ERG1c deactivation is significantly faster compared to ERG1a, leading to a reduction in current accumulation during repetitive firing (Heide et al., 2012). Because of this, ERG1c upregulation increases AP firing frequency and reduces spike frequency adaptation as seen in primary cortical neurons (Huffaker et al., 2009). Complementary fMRI data in schizophrenic patients with genetic variants associated with increased ERG1c show analogous hippocampus-prefrontal cortex disruption (Ren et al., 2020). In the context of drug treatment for schizophrenic patients, the antipsychotic Risperidone is a known ERG1c blocker and shows greater therapeutic efficacy in patients carrying genetic variants associated with upregulated ERG1c (Heide et al., 2016). Other analyses of schizophrenic patients treated with antidopaminergic drugs also showcase higher therapeutic success in patients with genetic variants causing increased expression of ERG1c (Apud et al., 2012). ERG1c is also linked to cognitive dysfunction, where ERG1c overexpression disrupts synaptic transmission and synapse formation between the hippocampus and prefrontal cortex in mice (Carr et al., 2016; Ren et al., 2020). ERG1c overexpression also disrupts behavioral working memory tasks while ERG1c suppression rescues behavior and promotes synapse formation (Carr et al., 2016). These data highlight ERG1c’s clinical relevance in neuronal disease as well as its potential as a therapeutic target.

### *KCNH2* Variants Cause Electrical Disturbances in Both the Brain and Heart

Inherited and acquired alterations in ion channel expression and function lead to alterations in electrical function, which ultimately provide substrates for both arrhythmias and seizures. While the phenotype of any given channelopathy may be primarily neuronal or cardiac, as many of these channels are expressed in both the brain and heart, they often include electrical disturbances in both the heart (arrhythmias) and brain (seizures) (Goldman et al., 2009; Johnson et al., 2009; Glasscock et al., 2010; Auerbach et al., 2013, 2016; Kalume et al., 2013; Anderson J. H. et al., 2014). As discussed above, *KCNH2*/K_v_11.1 is expressed in both the heart and brain. Despite *KCNH2*/K_v_11.1 being predominantly studied in the heart, due to its critical role in repolarization, it was initially cloned from a human hippocampal cDNA library (Warmke and Ganetzky, 1994). Furthermore, mutations in the drosophila *Kcnh2* homolog are associated with ether-induced seizure-like activity (*Ether-à-go-go dance*), and heat-induced seizures (Kaplan and Trout, 1969; Titus et al., 1997; Hill et al., 2019).

### Congenital Long QT Syndrome and Epilepsy

Long QT syndrome (LQT) is an arrhythmogenic cardiac disorder most notably associated with prolongation of the QT interval measured by a surface ECG (Moss, 2003). At least 17 distinct genes can trigger LQT [for review of LQT genetics see Skinner et al. (2019)]. LQT type 2 (LQT2) is caused by loss-of-function variants in the *KCNH2* gene that result in reduced cardiac I_Kr_, cardiomyocyte action potential prolongation, and QT_*c*_ prolongation on the surface ECG (Sanguinetti et al., 1995; Trudeau et al., 1995). LQT2 patients and animal models of LQT2 exhibit cardiac hyperexcitability, such as early after depolarizations in myocytes, and ectopic activity (e.g., premature ventricular complexes) (Brunner et al., 2008). LQT2 patients are prone to ventricular tachy-arrhythmias, such as torsades-de-pointes, and ultimately sudden cardiac death (Crotti et al., 2008).

LQT2-associated *KCNH2* variants cause either reduced ERG1 channel synthesis (Class 1), disruption in the intracellular transport/trafficking of the ERG1 protein to the cell membrane (Class 2), abnormal ERG1 channel gating (Class 3), or altered ERG1 permeability/selectivity (Class 4) (Delisle et al., 2004; Anderson C. L. et al., 2014; Figure 1). Most LQT2-linked variants reduce the number of channels on the membrane via Class 1/2 mechanisms; others are nonsense mutations, and the majority predict haploinsufficiency through nonsense-mediated RNA decay (Class 1 mechanism) (Splawski et al., 2000; Anderson et al., 2006; Gong et al., 2007; Stump et al., 2013; Anderson C. L. et al., 2014; Mehta et al., 2014, 2018). In a study analyzing 226 LQT2-associated *KCNH2* variants, 62% are nonsense mutations, 24% are in-frame insertion/deletion, 7% are splice site mutants, and 3% are in-frame ins/del in the *KCNH2* gene (Kapplinger et al., 2009). 32% of the variants resided in the transmembrane and pore-pore domains, 29% in the NH_2_ terminal (8% at the PAS/PAC domains), and 31% at the carboxy-terminus (8% at the CNBHD) (Kapplinger et al., 2009). Interestingly, mutations within the pore coincided with an increased risk of cardiac arrhythmia (Moss et al., 2002; Shimizu et al., 2009). A later report studying 167 *KCNH2* missense variants demonstrated that 76% of *KCNH2* mutations at the ERG1 pore totally abolished surface trafficking of co-expressed wildtype channels – a strictly dominant negative phenotype (Anderson C. L. et al., 2014). This strict dominant negative effect was not observed for mutations in other regions of the channel (Anderson C. L. et al., 2014).

There are multiple case studies of LQT2 patients with a history of epilepsy and seizure events (Omichi et al., 2010; Zamorano-Leon et al., 2012; Partemi et al., 2013, 2015; Li et al., 2016; Miyazaki et al., 2016; Zarroli and Querfurth, 2018; Figure 1 and Table 1). Using two LQT patient datasets, Johnson et al. (2009) and Auerbach et al. (2016), reported a higher prevalence of seizures (i.e., history of seizures/epilepsy or taking anti-anti-seizure medications, ASMs) in LQT2, compared to LQT1, LQT3, and LQT genotype-negative un-/related participants. *KCNH2* variants in the pore domain conferred the highest prevalence and risk of seizures (Auerbach et al., 2016). Among LQT patients with EEG evaluation, epileptiform activity, convulsive seizures, and an epilepsy diagnosis were documented at a rate 5-fold higher in LQT2 vs. all other types of LQT (Anderson J. H. et al., 2014). Additionally, LQT2 patients with interictal and ictal epileptiform activity in the temporal lobe has been reported (Anderson J. H. et al., 2014).

Epilepsy in LQT2 patients also appears to be independent of cardiac electrical dysfunction. Despite similar QT_c_ durations between LQT1 and LQT3 patients (Schwartz et al., 2001; Moss et al., 2007; Shimizu et al., 2009), LQT2 patients have the highest prevalence of seizures [12% LQT1, *n* = 432; 18% LQT2, *n* = 420; 9% LQT3, *n* = 113; 5% LQT^(–),^ *n* = 936] (Auerbach et al., 2016). Time-dependent adjusted risk assessments indicated that LQT2 patients are at the highest risk of seizures vs. all other groups (Auerbach et al., 2016). Further, there are multiple reports of epilepsy patients (including SUDEP) carrying LQT2-associated variants with minimal diagnosed cardiac electrical dysfunction (Keller et al., 2009; Anderson et al., 2012; Bagnall et al., 2016). Beta-adrenergic blockade reduces the time-dependent risk of arrhythmias (*p* = 0.004), but not seizures (*p* = 0.324) (Auerbach et al., 2016). Similar differences are seen when comparing only patients with both a history of seizure/epilepsy and prescribed ASMs (Auerbach et al., 2016). Collectively, these data suggest that cardiac electrical dysfunction is not a prerequisite for epileptogenesis in the context of *KCNH2* variants.

It is also important to recognize that there is an apparent bidirectional relationship between arrhythmias and seizures in LQT2. For example, a *KCNH2* variant provides a substrate for arrhythmias, which may result in cerebral hypoperfusion triggered seizures. Similarly, seizure-mediated autonomic dysfunction and central cardiorespiratory depression may provide triggers for arrhythmias (Nashef et al., 2007). This is particularly relevant as abrupt increases in sympathetic function (startle and stress) are triggers for arrhythmias in LQT2 (Schwartz and Priori, 2004). In a LQT2 patient, ventricular ectopy and the initiation of a near-lethal cardiac arrhythmia (*torsades-de-pointes*) was captured during a seizure (Omichi et al., 2010). Paroxysmal EEG slow waves were consistent with a potential underlying epileptic phenotype, and the coexistence of dual neuro-cardiac pathologies in LQT2 (Omichi et al., 2010). Furthermore, in a mouse model of LQT1 (*Kcnq1* mutation), there is a high prevalence and concordance of EEG abnormalities, seizures, autonomic instability, ECG abnormalities, and arrhythmias (Goldman et al., 2009).

Small molecules that increase terminal glycosylation or activators that increase the ERG1 channel’s open probability have demonstrated the capacity to restore normal channel trafficking and action potential duration (Ficker et al., 2005; Anderson C. L. et al., 2014; Sanguinetti, 2014). These drugs, however, are linked to drug-induced LQT (Spector et al., 1996). Recently, it was found that the use of blockers and activators in combination could increase the functional expression of ERG1 channels (Qile et al., 2019, 2020), raising the possibility that these treatments could be used together to increase channel trafficking to the plasma membrane. Nevertheless, these maneuvers need to be studied further, because there is a high possibility that increasing the mutant ERG1 channels will result in additional problems, such as changes in gating or permeability, which could worsen LQT2 or their impact on neuronal function.

### LQT2 and Sudden Unexpected Death in Epilepsy

Patients with epilepsy are at a 24-fold increased risk of sudden death compared to the general population (Ficker, 2000). The leading cause of epilepsy-related death is SUDEP (1–6 cases per 1,000 patient/years), and is 2nd among all neurological diseases in years of potential life lost (Thurman et al., 2014). SUDEP is defined as sudden unexpected, witnessed or unwitnessed, non-traumatic, non-drowning death, with or without evidence of a seizure in an individual with epilepsy, excluding status epilepticus, and postmortem examination does not indicate a toxicological or apparent cause of death (Nashef et al., 2012). While the mechanisms for SUDEP remain unknown, cardiac arrhythmias, respiratory disturbances, cerebral hypoperfusion, failed arousal, and autonomic abnormalities are the proposed mechanisms for SUDEP (Devinsky et al., 2016).

In addition to several epilepsy-related gene variants, post-mortem exome sequencing analyses of 61 SUDEP cases identified a high prevalence of genes linked to arrhythmias (21%, *n* = 13), especially LQT (Bagnall et al., 2016). *KCNH2* was among the top 30 genes with the greatest number of rare variants in SUDEP vs. controls (Bagnall et al., 2016). There was a higher prevalence of loss-of-function (3-fold) and rare variant (11-fold) *KCNH2* variants in SUDEP cases vs. epilepsy controls (Soh et al., 2021). In a separate study, 13% (6:48) of SUDEP cases had non-synonymous *KCNH2* and *SCN5A* (Na^+^ channel) variants linked to LQT2 and LQT3, respectively (Tu et al., 2011).

Epilepsy is associated with chronic and peri-ictal altered cardiac electrical function, which include QT_c_ prolongation, T-wave alternans, and conduction defects (Surges et al., 2010a,b,c; Surges and Walker, 2010; Strzelczyk et al., 2011; Auerbach et al., 2013; van der Lende et al., 2015; Verrier et al., 2016, 2020; Shmuely et al., 2020). There is a significantly higher prevalence and risk of arrhythmias in LQT2 patients with vs. without a history of seizures (Auerbach et al., 2016). Thus, a *KCNH2* variant linked to LQT2, and epilepsy-mediated electrical remodeling, likely synergistically increase the risk of arrhythmias and SUDEP (Bleakley et al., 2020).

### Acquired LQT

While the above discussion focused on LQT2 variants altering cardiac and neuronal electrical function, many medications have off target ERG1 blocking properties. ERG1 is highly susceptible to open channel block (Vorperian et al., 1996; Mohammad et al., 1997; Drolet et al., 1999; Zhang et al., 1999; Zhou et al., 1999; Sanchez-Chapula et al., 2002; Rajamani et al., 2006; Sanguinetti and Tristani-Firouzi, 2006), and off-target ERG1 block is the primary cause of acquired long QT syndrome (Sanguinetti et al., 1995). Like LQT2 patients, patients with acquired long QT syndrome have an increased incidence of cardiac arrhythmias and sudden cardiac death (Sanguinetti et al., 1995). The FDA mandates that all lead compounds must be screened for off target ERG1 blockade and QTc prolongation, prior to approval (Food and Drug Administration, HHS, 2005). Many ASMs (e.g., phenytoin and lamotrigine) exert off-target I_Kr_ blocking properties at therapeutic free plasma concentrations (Danielsson et al., 2003, 2005), and would be expected to impact neuronal ERG channels. This is particularly relevant in LQT2 patients, as ASMs, particularly sodium channel blocking ASMs, result in an increase in the recurrent risk of cardiac events (Auerbach et al., 2018). Surprisingly, the impact of off-target ERG1 channel block on neuronal function has not been explored in detail.

Many previous studies have increased our understanding of the molecular mechanisms of ERG1 channel block. These studies identified several key residues as critical drug binding determinants: Thr623 and Ser624 at the inner mouth of the pore helix, and Tyr652 and Phe656 of the S6 helix (Lees-Miller et al., 2000; Mitcheson et al., 2000; Sanchez-Chapula et al., 2003; Fernandez et al., 2004; Kamiya et al., 2006; Durdagi et al., 2010). Access to these binding sites occurs following channel activation (open channel block) (Sanchez-Chapula et al., 2002; Sanguinetti and Tristani-Firouzi, 2006). A subset of ERG1 channel blockers display preferential binding to the ERG1 inactivated state (modulated receptor hypothesis) (Yang et al., 2004). *KCNH2* mutations that impair or remove ERG1 inactivation reduce the affinity of numerous clinically relevant drugs such as cisapride, quinidine, and dofetilide (Ficker et al., 1998; Lees-Miller et al., 2000; Yang et al., 2004; Perrin et al., 2008). Similarly, modulating external cations to disrupt inactivation also reduces drug binding (Wang et al., 1997b; Numaguchi et al., 2000). Last, ERG1 subunit abundance, which modifies channel gating, was also shown to mediate the efficacy of block (Sale et al., 2008; Abi-Gerges et al., 2011; El Harchi et al., 2018). Thus, the dynamic nature of the drug-binding site of ERG1 channels is dependent on both the channel’s conformational changes and subunit stoichiometry. Additional positions at the extracellular regions and the outer mouth of the selectivity filter, can bind ERG inhibitors but are far less dependent upon channel gating (Korolkova et al., 2002; Milnes et al., 2003; Zhang et al., 2003, 2007; Liu et al., 2012; Yu et al., 2014). It is highly likely that these characteristics of ERG1 block are conserved in ERG2 and ERG3 given their similar gating and near perfect homology at the pore and S6 helices (Figure 7; Shi et al., 1997).

### Therapeutic Potential of Neuronal ERG1

Therapeutics with off-target inhibitory effects on ERG1 have shown enhanced efficacy in treating schizophrenic patients when ERG1c was upregulated, such as Risperidone (Heide et al., 2016) and antidopaminergic drugs (Apud et al., 2012). Targeted ERG inhibition to treat neuronal dysfunction would promote life-threatening cardiac arrhythmia and is not a viable therapeutic strategy. Activators of ERG1 and its orthologs, ERG2 and ERG3, may represent a more viable treatment for neuronal diseases, such as epilepsy.

ERG1 activators, including RPR260243, ICA105574, and PD-118057 (Kang et al., 2005; Zhou et al., 2005; Gerlach et al., 2010), enhance ERG1 current by inhibiting channel inactivation, promoting activation, or delaying deactivation (Sanguinetti, 2014). ERG1 activators that disrupt inactivation can dramatically shorten the action potential duration in cardiomyocytes (Bentzen et al., 2011; Zhang H. et al., 2012; Yu et al., 2016; Perry et al., 2019; Qile et al., 2019), but are associated with an unexpected increased incidence of arrhythmia *in vivo* (Bentzen et al., 2011). One report suggested that small molecules that disrupt ERG channel inactivation share a non-selective pharmacophore (Schewe et al., 2019). NS-1643, which disrupts ERG inactivation (Casis et al., 2006; Bilet and Bauer, 2012), was shown to reduce epileptogenesis in mice (Xiao et al., 2018). Unfortunately, ERG1 plays a minimal role in murine cardiac repolarization, so this finding cannot predict potential pro-arrhythmic effects in humans. It does, however, highlight the therapeutic potential of ERG activators in the brain. Harley et al. (2016), demonstrated that selective disruption of the ERG1 PAS domain increased ERG currents and shortened the action potential duration in human cardiomyocytes, suggesting that the ERG PAS domain could work as an alternative target to enhance ERG current amplitude. ERG1 activators that slow channel deactivation have shown limited effects on cardiac repolarization (Zhang H. et al., 2012; Shi et al., 2020a), but could be a useful tool to dampen neuronal excitability. It should be acknowledged that the high homology between orthologs suggests that the development of any ERG1-specific modulator would be challenging.

## Discussion

The ERG family of channels serve to stabilize the neuronal resting membrane potential, suppress hyperexcitability, and impact the spike frequency adaptation to varying degrees throughout the mammalian central nervous system (Chiesa et al., 1997; Sacco et al., 2003; Pessia et al., 2008; Hardman and Forsythe, 2009; Babcock and Li, 2013). I_K(ERG)_ blockade promotes neuronal hyperexcitability, as seen by increased spontaneous activity, spike frequency, plateau potential, and decreased threshold for action potential firing (Sacco et al., 2003; Pessia et al., 2008; Hardman and Forsythe, 2009; Ji et al., 2012). The surprisingly high incidence of epilepsy and seizure in the context of LQT2-associated *KCNH2* variants highlights the ability of ERG1 channels to modulate neuronal behavior. As ERG1 is expressed across multiple neuronal cell types, it is difficult to identify the mechanism by which *KCNH2* variants promote neuronal dysfunction. ERG1 is, however, important in neuronal physiology and deserves more study.

## Author Contributions

FS-C, EJ-V, DA, and DJ collectively wrote and edited the manuscript. All authors contributed to the article and approved the submitted version.

## Conflict of Interest

The authors declare that the research was conducted in the absence of any commercial or financial relationships that could be construed as a potential conflict of interest.

## Publisher’s Note

All claims expressed in this article are solely those of the authors and do not necessarily represent those of their affiliated organizations, or those of the publisher, the editors and the reviewers. Any product that may be evaluated in this article, or claim that may be made by its manufacturer, is not guaranteed or endorsed by the publisher.
